# Assessment of metal extraction from e-waste using supported IL membrane with reliable comparison between RSM regression and ANN framework

**DOI:** 10.1038/s41598-024-54591-y

**Published:** 2024-02-16

**Authors:** Alireza Hemmati, Mehdi Asadollahzadeh, Rezvan Torkaman

**Affiliations:** 1 School of Chemical, Petroleum and Gas Engineering, Iran University of Science and Technology, P.O. Box: 16765-163, Tehran, Iran; 2 Nuclear Fuel Cycle Research School, Nuclear Science and Technology Research Institute, P.O. Box 11365-8486, Tehran, Iran

**Keywords:** Indium ions, Artificial neural network, Flat sheet supported liquid membrane, Experimental design, Response surface methodology, Mathematics and computing, Chemical engineering

## Abstract

Recently, efficient techniques to remove indium ions from e-waste have been described due to their critical application. This paper illustrates the recovery of indium ions from an aqueous solution using a liquid membrane. CyphosIL 104 described the excellent potential for the extraction of indium ions. Evaluation of the five process parameters, such as indium concentration (10–100 mg/L), carrier concentration (0.05–0.2 mol/L), feed phase acidity (0.01–3 mol/L), chloride ion concentration (0.5–4 mol/L) and the stripping agent concentration (0.1–5 mol/L) were conducted. The interactive impacts of the various parameters on the extraction efficiency were investigated. The response surface methodology (RSM) and artificial neural network (ANN) were employed to model and compare the FS-SLM process results. RSM model with a quadratic equation (R^2^ = 0.9589) was the most suitable model for describing the efficiency. ANN model with six neurons showed a prediction of extraction efficiency with R^2^ = 0.9860. The best-optimized data were: 73.92 mg/L, 0.157 mol/L, 1.386 mol/L, 2.99 mol/L, and 3.06 mol/L for indium concentration, carrier concentration, feed phase acidity, chloride ion concentration, and stripping agent concentration. The results achieved by RSM and ANN led to an experimentally determined extraction efficiency of 93.91%, and 94.85%, respectively. It was close to the experimental data in the optimization condition (95.77%). Also, the evaluation shows that the ANN model has a better prediction and fitting ability to reach outcomes than the RSM model.

## Introduction

**Figure 1:**
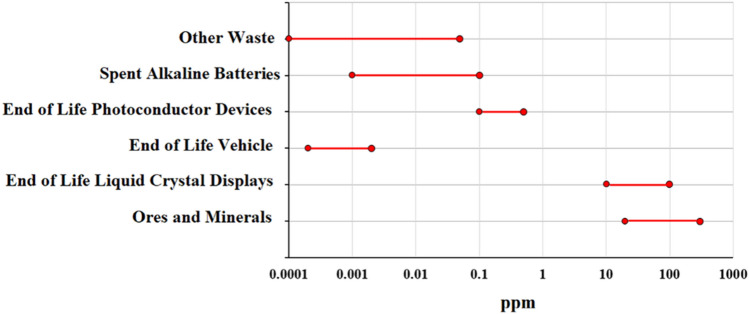
Indium concertation in various materials.

Indium is one of the essential metals in the electronics industry. It usually appears in sulfide ores such as zinc (sphalerite), lead (galena), copper (polymetallic), and tin (cassiterite and stannite). Asphalrite is the most critical ore containing indium^1,2^. In the primary process, indium remains in the leaching residue of the leached concentrate, the central part of which is iron compounds^3,4^. Therefore, it is necessary to dissolve the leaching residue in the hot and concentrated sulfuric acid solution^5,6^. This method is suitable for a leaching solution in which the indium concentration is enough for separation. However, suppose the concentration of indium in the zinc leaching residue is deficient^7^. In that case, therefore, its concentration in the resulting leaching solution is low. As shown in Fig. 1, these low concentrations are observed in the secondary sources such as the spent liquid crystal displays^8–10^, vehicles, photoconductor devices^11^, and spent alkaline batteries^12–16^.

Thus, there is a need for selective methods to extract indium and other heavy metals^17–21^. The process of liquid–liquid extraction plays a crucial role in various industries for separating substances^22–24^. The worldwide focus on solvent extraction for purifying and separating valuable metals has increased due to its extensive use in major technological sectors^25–27^. Over the past ten years, there has been a notable advancement in the comprehension of the functioning of equipment used in solvent extraction^28–30^. However, there are certain domains that still require further exploration of fundamental principles in physics, as well as the creation of models and prediction tools to elucidate these occurrences^31–33^. These areas encompass a deeper understanding of the mechanisms responsible for the fragmentation and merging of droplets within a shear field, specifically when considering mass transfer and the influence of surfactants^34^.

Solvent extraction is widely used in various difficult scenarios that require extreme phase ratios and minimal solvent loss^35^. One such application is the extraction of lithium from brines and the recovery of materials from rechargeable batteries. In addition, due to stricter environmental regulations, the treatment of low-concentration streams that were previously disposed of has become necessary, often involving extreme phase ratios^36^.

Indium extraction is done with commercial extractants such as D2EHPA^37–39^, TOA, TBP^40^.

Today, the literature has also reported the use of ionic liquids (ILS) or deep eutectic solvents for the separation of this element^41–45^. ILS have been widely adopted as greener alternatives to traditional organic solvents for solvent extractions. Their utilization aims to improve the separation efficiency of established extractants. This shift in preference is primarily due to the advantageous characteristics of ILs. Alquacil and co-authors illustrated the extraction of In(III) from H_2_SO_4_ solution with the synthesized ionic liquid. Feed acidity is the main factor in the extraction procedure^46^. In another study, the obtained ionic liquid (A324H^+^)(Cl^−^) from acid–base chemical reaction was used for In(III) recovery with the anion exchange mechanism^47^. Kashyap and Taylor presented the findings that a maximum indium concentration of 0.121 g/L was achieved through multiple step leaching. The influence of temperature, acid concentration, liquid–solid ratio (L/S), and the impact of adding oxalic acid on the leaching process were also examined and discussed^48^. Grigorieva and co-workers demonstrated that incorporating proton-donor additives (HA) into the D2EHPA solution is an effective method for achieving a high stripping efficiency of indium. These extraction systems, which consist of D2EHPA and 2-ethylhexanol, can effectively recover indium from different industrial solutions^49^. Liu and colleagues detailed a method for separating Cu, In, Ga, and Se from thin-film solar panels. The extraction process involved using di-(2-ethylhexyl) phosphoric acid to transfer the elements into the organic phase, while Cu and Ga were retained in the aqueous phase^50^. Gao and co-workers discovered that the extraction method using a combination of hydrophobic ionic liquid, DE2HPA extractant, and tributyl phosphate was able to extract only indium ions. Furthermore, indium ions could be effectively separated using HCl^51^. Li and colleagues conducted research on the P204-solvent-impregnated resin employed in the experimental procedure. This study showcases a novel and eco-friendly approach to separate and purify Ga and In from wastewater^52^.

In addition to selecting an extractant with a high separation factor, the possibility of using a new process with a high recovery stage is also a method mentioned as intensifying the process by reducing the consumption of energy and the increment in efficiency. The T-type microreactor was used to recover this element from other impurities with D2EHPA extractant. The separation factor showed a higher value (β_In/Fe_ ~ 18,720) in this structure compared to solvent extraction (β_In/Fe_ ~ 276)^53^. The efficiency increment is developed by using non-aqueous solvent extraction. This process extracts indium ions with Cyphos IL 101 and Alamine 336 diluted in toluene as the organic phase. The extraction with high efficiency of 98% is carried out from the ethylene glycol phase to the organic phase in the mixer-settler^54^. Also, among the extraction methods, it is observed that the membrane processes are very efficient in processing materials with low concentrations^55–57^. This method is associated with low energy consumption, solvent consumption in one stage of extraction and stripping. Liquid membrane is a highly effective method for separation due to its selectivity, single-stage operation, and efficacy. The field of liquid membranes has garnered much attention and excitement in research, particularly in the realm of liquid–liquid and gas–liquid separation processes^58^. In the study of Meng and co-workers, polymer inclusion membrane including D2EHPA was used for In(III) recovery. The selective separation factor higher than 34.33 is obtained under the first-order kinetics^59^. The membrane oil–water extractor was used to extract indium. The removal efficiency of 99% is achieved with D2EHPA 0.08 M, but below 85% is observed with the traditional procedure^60^. The extraction of indium from waste liquid crystal displays (LCDs) was conducted through the utilization of ultrasound leaching and liquid membrane. The indium recovery rate can reach approximately 80%, while the final product solution can achieve an indium purity of nearly 100%. These techniques prove to be highly effective in efficiently reclaiming indium from waste LCDs^61^.

The novel procedures for the investigation of the main parameters of indium recovery with liquid membrane approach are scarce. Therefore, this work illustrated the indium extraction by using response surface methodology (RSM), and artificial neural networks (ANN) with flat sheet supported liquid membrane (FS-SLM). The extraction of indium from the aqueous phase to the organic phase using a diluted solution of CyphosIL 101 in kerosene was examined using both RSM and ANN methods. The highest level of indium recovery efficiency was achieved at the optimum conditions, which included a 4 mol/L acidity level in the aqueous phase, an indium concentration of 197.79 ppm, an ionic liquid concentration of 0.009 mol/L, and an aqueous to organic phase ratio of 1.58 mol/L^62^.

The RSM approach is widely known as a potential technique to estimate the relationship between input variables and responses. In this approach, the procedure calculates the main, interaction, and independent parameters to predict results and propose an explicit mathematical equation to describe the relationship between variables and answers. Finally, the optimization data was reported based on the desirable goal. The advantage of using a CCD is the possibility of extracting more information from the analysis of this design and performing fewer optimization experiments and less repetition of experiments, making the implementation of this method convenient and more accessible. Also, ANN was employed to evaluate data. The limitation of RSM is that it cannot control the impact parameters. ANN is soft computing technique that involve studying processes by changing the network’s weights to generate the required response. However, no detailed knowledge of the physical/chemical processes that affect the system is required. The new procedure is illustrated by the use of CyphosIL 104 and FS-SLM technique with RSM and ANN approaches. The evaluation of the interactive parameters for indium efficiency was described for the first time in this research work.

## Experimental

### Materials

In experimental works, trihexyl(tetradecyl)phosphonium bis(2,4,4-trimethylpentyl) phosphinate (Cyphos IL 104, Sigma-Aldrich, C_48_H_102_O_2_P_2_, > 90%, CAS-Number: 465527-59-7) was used as the extractant phase diluted in kerosene (Mixture of hydrocarbons, > 90%, CAS-Number: 64742-48-9). Indium nitrate from Sigma-Aldrich (> 99.9%, In(NO_3_)_3_·xH_2_O, CAS-Number: 207398-97-8) was used in the preparation of feed phase. The stripping solution was prepared from the nitric acid concentration (Merck company, HNO_3_, 65% > , CAS-Nummer:7697-37-2). The hydrophobic membrane from Merck Millipore (total diameter ~ 47 mm, effective diameter ~ 35 mm, thickness ~ 150 μm, pore size ~ 0.22 μm, and porosity 85%) was used in all experiments.

### Experimental setup

**Figure 2:**
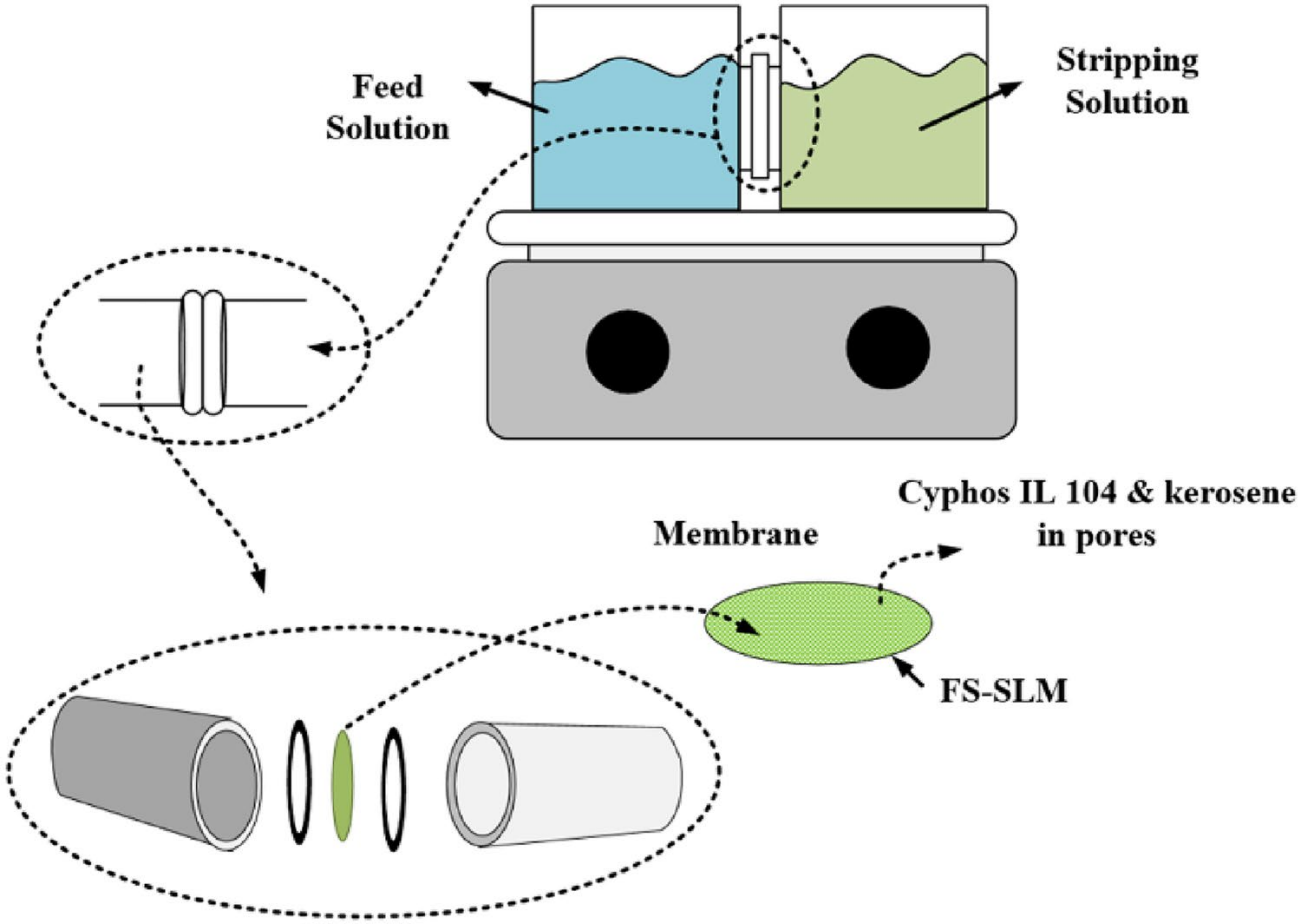
Schematic of supported liquid membrane.

The membranes were immersed in the organic phase solution (ionic liquid diluted in kerosene) overnight so that all their pores were filled with the organic phase and could be used as a carrier phase. Two glass containers with 20 mL volume for feed and stripping solutions and the holding of FS-SLM between the glass flanges was used in the experiments, as shown in Fig. 2. The indium transport was obtained with UV–visible spectrophotometer (Shimadzu UV-1800, Japan) of both phases. The transport efficiency (%E) at any given time was obtained as:

1 $$ \% E = \frac{{(C_{0} - C_{t} )}}{{C_{0} }} \times 100 $$

C_0_ and C_t _are the concentration of indium ion in the zero time and after time t, respectively.

### Design parameters

The ranges of the selective parameters base on the initial experiments and according to the central composite design approach is shown in Table 1. Fifty runs were obtained by the Design-EXPERT 8.0 software to objective data (extraction efficiency) with an FS-SLM setup at different conditions.

**Table 1:** Actual and coded values for selective parameters.

Factors	Parameters	Units	Coded low	Coded high
A	HNO_3_ concentration in the feed phase	mol/L	-1 ↔ 0.01	+ 1 ↔ 3
B	Indium concentration in the feed phase	mg/L	-1 ↔ 10	+ 1 ↔ 100
C	Cyphos IL 104 concentration	mol/L	-1 ↔ 0.05	+ 1 ↔ 0.2
D	Stripping concentration	mol/L	-1 ↔ 0.1	+ 1 ↔ 5
E	Chloride concentration	mol/L	-1 ↔ 0.5	+ 1 ↔ 4

A set of preliminary experiments and initial objectives were utilized to determine the range of parameters displayed in this table.

Equation (2) was used for the description of data:

2 $$ \% E = \alpha_{0} + \sum\limits_{i = 1}^{k} {\alpha_{i} } X_{i} + \sum\limits_{i = 1}^{k} {\alpha_{ii} } X_{i}^{2} + \sum {\sum\limits_{i < j}^{k} {\alpha_{ij} } } X_{i} X_{j} $$

### Artificial neural network approach

**Figure 3:**
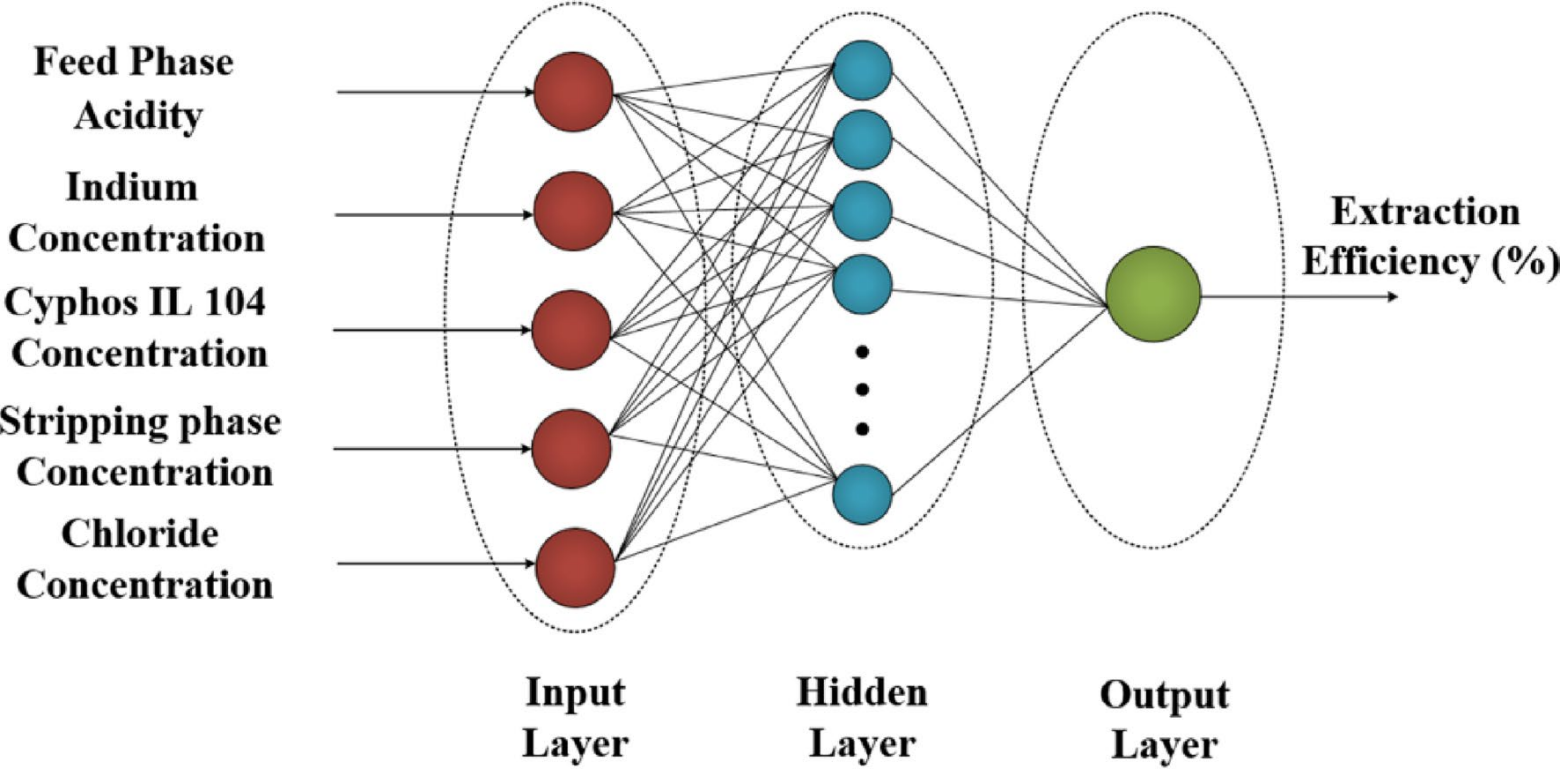
Structure of artificial neural network.

The schematic model of the artificial neural network with three layers is described in Fig. 3. This model with input, hidden and output layers was used instead of the polynomial regression approach. The main procedure is the selective data for the numbers of neurons in hidden layer.

The feed-forward backpropagation was used to model description with Matlab software. Five input factors was selected in input layer, including, feed phase acidity, indium concertation, stripping phase concentration, Cyphos IL 104 concentration, and chloride ion concentration. The extraction efficiency was chosen as the objective data in the output layer. The Levenberg–Marquardt with Tansig transfer function is applied in the model description. Three groups (training (60%), testing (20%), and validation (20%) was used in the division of data for the modeling.

The statistical parameters for the evaluation of two models are as follows:

Root mean square error:

3 $$ RMSE = \sqrt {\frac{{\sum\nolimits_{i = 1}^{N} {\left( {\Pr edicted_{i} - Actual_{i} } \right)^{2} } }}{N}} $$

Average absolute relative error:

4 $$ AARE = \frac{{\sum\nolimits_{i = 1}^{N} {\left| {\frac{{\left( {\Pr edicted_{i} - Actual_{i} } \right)}}{{Actual_{i} }}} \right|} }}{N} \times 100 $$

## Results and discussion

### RSM Procedure

The CCD technique with quadratic equation was utilized to the experiments (see Table 2). This Table showed the observed data including R^2^ ~ 0.9589, adjusted R^2^ ~ 0.9306, and predicted R^2^ ~ 0.8725 for the quadratic model. The described equation is as follows:

5 $$ \begin{aligned} \% E & = { - 256}{\text{.04 + 120}}{.87} \times {\text{A + 0}}{.73} \times {\text{B + 1334}}{.42} \times {\text{C + 53}}{.85} \times {\text{D + 9}}{.34} \times {\text{E}} \hfill \\ & \quad { - 0}{\text{.28}} \times {\text{A}} \times {\text{B}}{ - 304.66} \times {\text{A}} \times {\text{C}}{ - 2.44} \times {\text{A}} \times {\text{D + 2}}{.63} \times {\text{A}} \times {\text{E + 0}}{.87} \times {\text{B}} \times {\text{C}} \hfill \\ & \quad  { - 0}{\text{.04}} \times {\text{B}} \times {\text{D + 0}}{.08} \times {\text{B}} \times {\text{E + 12}}{.66} \times {\text{C}} \times {\text{D + 7}}{.35} \times {\text{C}} \times {\text{E + 1}}{.59} \times {\text{D}} \times {\text{E}} \hfill \\ & \quad { - 19}{\text{.21}} \times {\text{A}}^{2} { - 0}{\text{.004}} \times {\text{B}}^{2} { - 2159}{\text{.01}} \times {\text{C}}^{2} { - 8}{\text{.94}} \times {\text{D}}^{2} { - 3}{\text{.37}} \times {\text{E}}^{2} \hfill \\ \end{aligned} $$

**Table 2:** Details of various models to the prediction of experimental data.

Source	Sequential p-value	Lack of fit p-value	Std. dev.	R^2^	Adjusted R^2^	Predicted R^2^	PRESS	
Linear	< 0.0001	0.0034	15.12	0.5813	0.5337	0.4742	12,629.64	
2FI	0.6956	0.0025	15.61	0.6549	0.5027	0.4801	12,488.40	
Quadratic	< 0.0001	0.4779	5.83	0.9589	0.9306	0.8725	3062.49	Suggested
Cubic	0.3650	0.5236	5.54	0.9821	0.9373	− 0.0531	25,297.98	Aliased

In the above equation, A, B, C, and D is feed phase acidity, indium concentration, carrier concentration, stripping agent concentration and chloride ion concentration, respectively. The increment and the decrement behavior is described with the positive and negative sign in the above equation.

**Figure 4:**
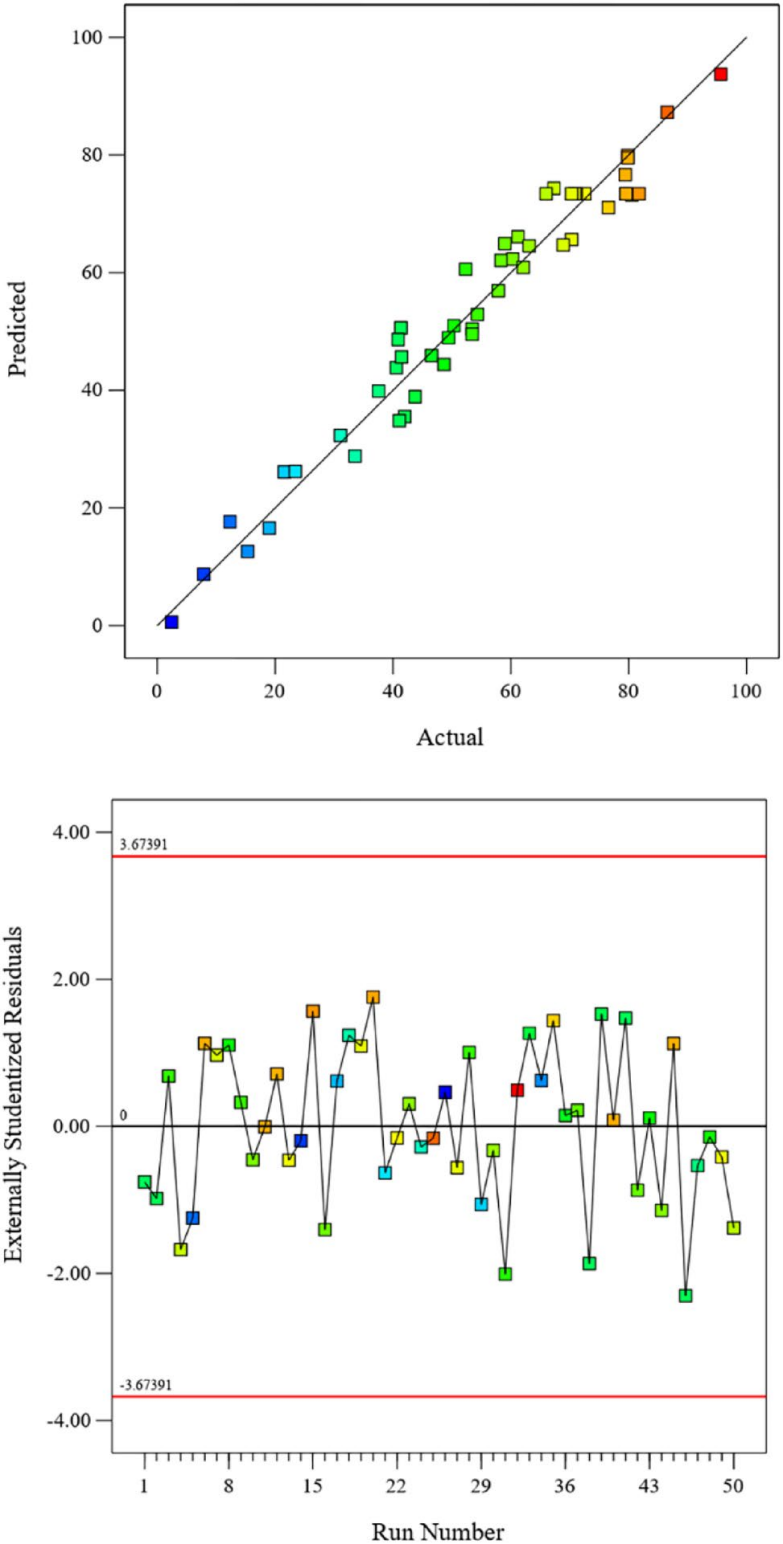
Correlation between actual and predicted values based on RSM approach with externally studentized residuals from the quadratic model.

The analysis of variance is described in Table 3 (ANOVA Table). The significant data are the F-value ~ 33, lack of fit for F-value ~ 1.11. Also, the comparison between the experimental data and the model data is shown in Fig. 4. The line behavior in this figure with the slope of one and ANOVA data is shown that the agreement between the selected input variables and the object function.

**Table 3:** ANOVA for indium recovery in FS-SLM system.

Source	Sum of squares	df	Mean square	F-value	p-value	
Model	23,035.33	20	1151.77	33.86	< 0.0001	Significant
A	1482.84	1	1482.84	43.60	0.0342	
B	198.59	1	198.59	5.84	0.0122	
C	8067.79	1	8067.79	237.21	0.0645	
D	2808.52	1	2808.52	82.58	0.0768	
E	1406.47	1	1406.47	41.35	< 0.0001	
AB	353.25	1	353.25	10.39	0.0031	
AC	1166.93	1	1166.93	34.31	< 0.0001	
AD	79.76	1	79.76	2.35	0.1365	
AE	47.48	1	47.48	1.40	0.2470	
BC	8.61	1	8.61	0.2532	0.6186	
BD	15.24	1	15.24	0.4479	0.5086	
BE	43.48	1	43.48	1.28	0.2675	
CD	5.41	1	5.41	0.1591	0.6929	
CE	0.9316	1	0.9316	0.0274	0.8697	
DE	46.80	1	46.80	1.38	0.2503	
A^2^	3201.06	1	3201.06	94.12	< 0.0001	
B^2^	95.72	1	95.72	2.81	0.1042	
C^2^	256.12	1	256.12	7.53	0.0103	
D^2^	5000.05	1	5000.05	147.01	< 0.0001	
E^2^	184.74	1	184.74	5.43	0.0269	
Residual	986.32	29	34.01			
Lack of fit	766.29	22	34.83	1.11	0.4779	Not significant
Pure error	220.03	7	31.43			
Cor total	24,021.65	49

### 3D curve for extraction efficiency

**Figure 5:**
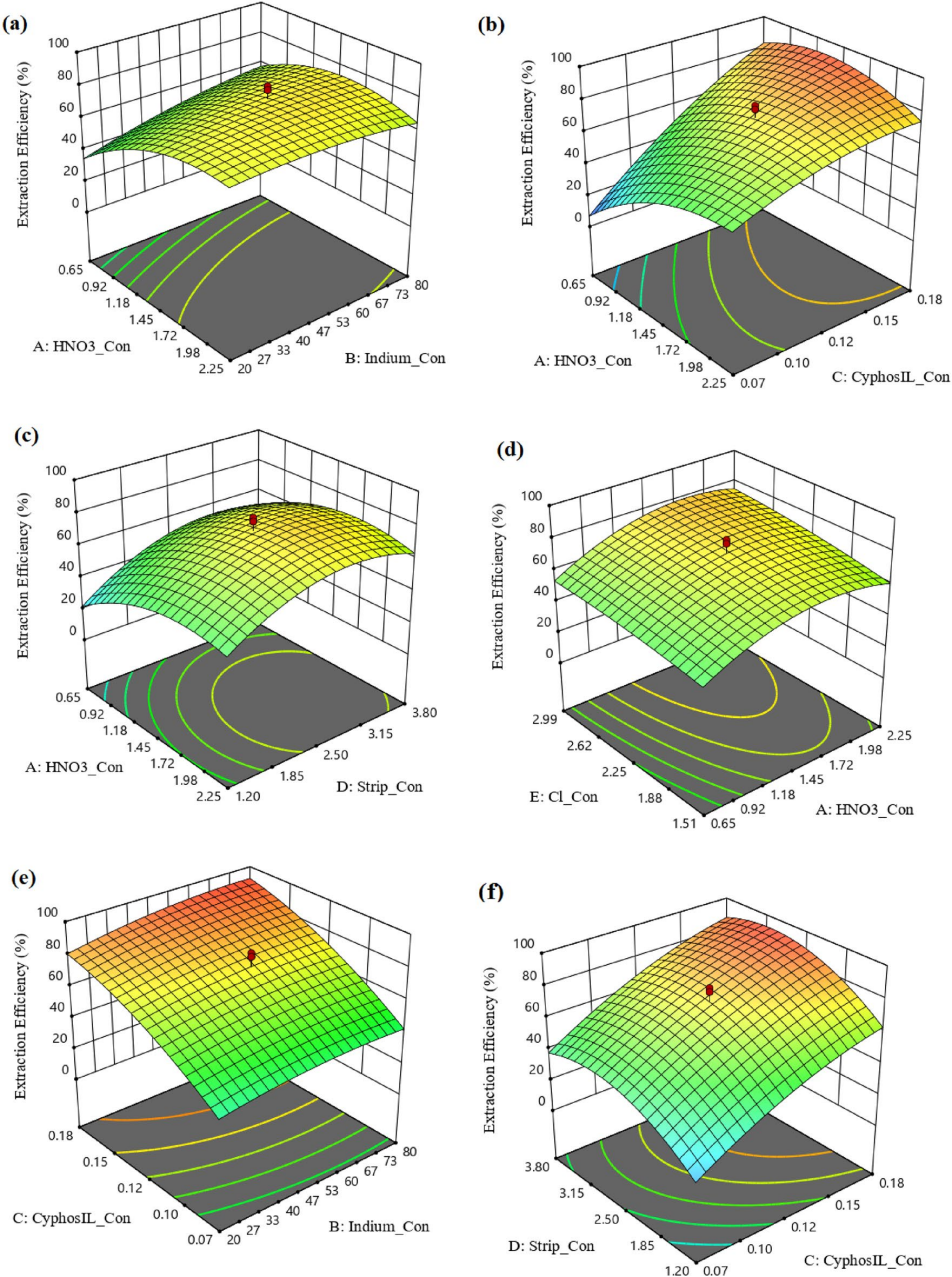
3D Plots of various factors on the extraction efficiency (**a**) impact of A and B factors; (**b**) impact of A and C factors; (**c**) impact of A and D factors; (**d**) impact of A and E factors; (**e**) impact of B and C factors; (**f**) impact of C and D factors.

The response surface for the evaluation of parameters for the indium recovery is shown in Fig. 5.

The indium(III) extraction from nitric acid solution was illustrated using 0.125 mol/L Cyphos IL 104 in kerosene, 2.55 mol/L of stripping phase, 2.25 mol/L of [Cl^−^], as shown in Fig. 5a. The indium(III) extraction at 0.01 mol/L HNO_3_ was obtained equal to 18.98%, and it incremented to 73.15% at 1.5 mol/L acidity concentration and decreased up to 3.0 mol/L HNO_3_. The hydrated cationic species In(H_2_O)_6_^+3^ at low acidity is described in the literature data. The extraction in the low acidity is related to the competition of indium ions with acid extraction. Also, the driving force from feed to the stripping phase decreases with the high values for HNO_3_ in the feed phase. Increasing the concentration of indium ions is associated with increasing the driving force for the reaction and transfer, so a linear trend in the system is achieved by raising the indium ions in the feed phase.

To enhance the impact of feed phase acidity and Cyphos IL 104 extractant was illustrated in Fig. 5b. The feed phase containing 55 mg/L of In(III), 2.55 mol/L of stripping phase, 2.25 mol/L of [Cl^−^] was used in the experiments. Changes in the effect of feed phase acidity in this system are similar to the previous diagram, and the extraction efficiency increases about 60% by changing the concentration from 0.05 to 0.2 mol/L of Cyphos IL 104. Because the transport carrier is a function of ionic liquid concentration, and when it is higher, the tendency to form a complex and penetrate is higher.

The simultaneous effect of acidity of feed and stripping phases is shown in Fig. 5c. The trend of Gaussian changes in the curve indicates that the transient driving force can reach the maximum efficiency by decreasing one parameter and increasing the other parameter. But at low acidity concentrations, the extraction rate is very low, and the possibility of favorable competition is low due to the tendency for extraction and reaction. This competition begins with the increment in the acidity of the feed and stripping phases. The high acidity of the feed phase (3 mol/L) creates a lower driving force for extraction, and the extraction percentage decreases. The acidity of the stripping phase is also associated with an increase. But at high concentrations, competition increases, which can lead to a reduction in the desired transfer. The impact of [Cl^−^] concentration on the extraction efficiency was investigated from 0.4 to 5 mol/L with NaCl solution. The efficiency enhances with the higher values for [Cl^−^] concentration due to the participation of chloride ions in the formation of the complex, as shown in Fig. 5d.

In Fig. 5e, the same behavior of the other parameters changes in the constant acidity of the feed phase has been reported. In Fig. 5e, the positive effects of both parameters are shown, and positive incremental changes by changing all parameters indicated that the process of ion diffusion, complex formation, and breakage rate of the complex in the selected interval increases and is accompanied by an increase in extraction percentage. The results in Fig. 5f showed the main effect of carrier concentration for complex formation and the increment in maximum efficiency.

**Figure 6:**
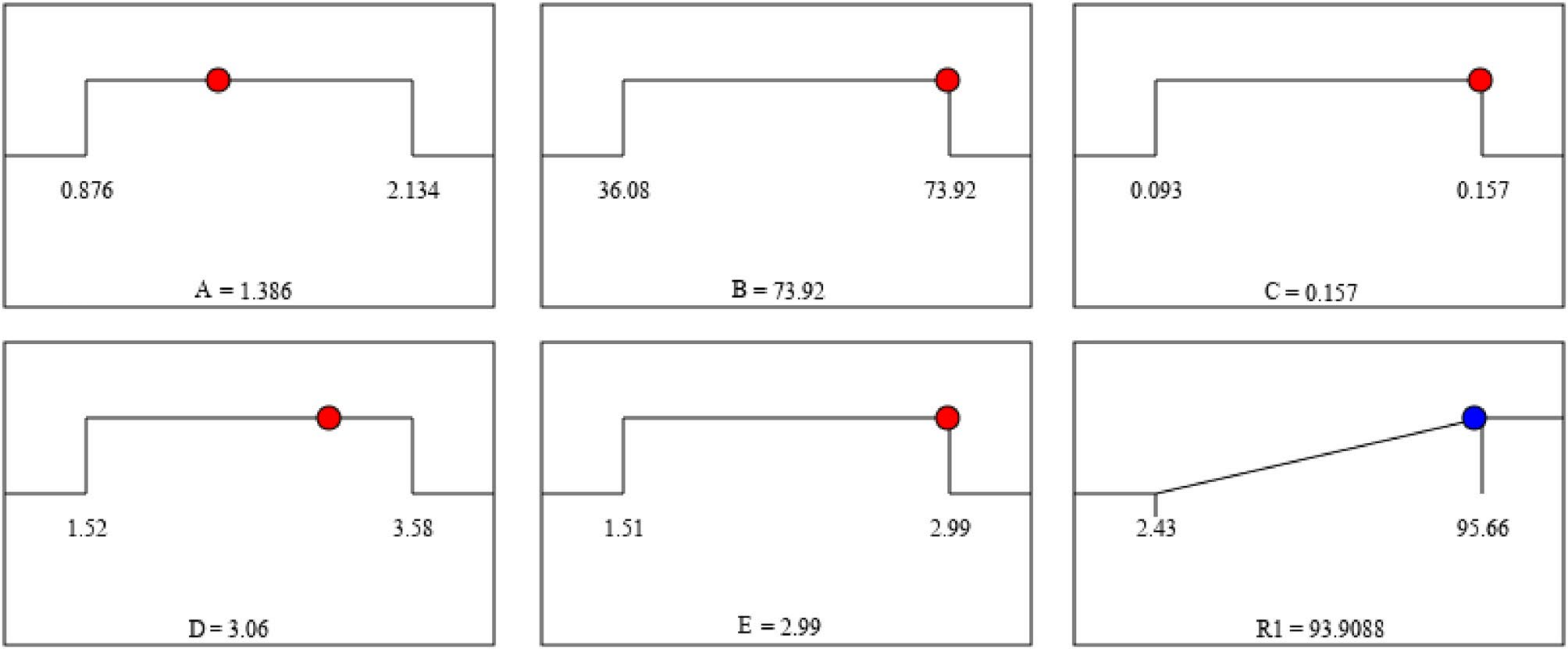
Profile of optimum condition for each variable to reach maximum extraction efficiency.

The optimization procedure was obtained according the selected data, as shown in Fig. 6. The efficiency of indium in the optimal condition was 93.91% under 1.386 mol/L feed phase acidity, 73.92 mg/L indium(III), 0.157 mol/L Cyphos IL 104 in FS-SLM, 3.06 mol/L of stripping phase, and [Cl^−^] concentration of 2.99 mol/L. The results from experimental work was 95.77% with the low deviation from the predicted results in the software.

In the aqueous solution, different chloro species of Indium are present, and their composition changes from being cationic to neutral to anionic as the acidity of the solution increases. The primary species of indium shift from InCl_2_^+^ and InCl^2+^ at less than 1 M HCl to a neutral InCl_3_ species between 1 and 6 M HCl. At higher HCl concentrations, singly and doubly charged anionic species InCl_4_^−^ and InCl_5_^2−^ become dominant. The current investigation focuses on a solution with 1.386 mol/L feed phase acidity and [Cl^−^] concentration of 2.99 mol/L from the optimum condition where the neutral InCl_3_ species is the most prevalent. Previous studies have suggested a mechanism involving adduct formation for extracting In(III) from an HCl solution using Cyphos IL 104^43,63^.

6 $$ In_{aq}^{ + 3} + 3Cl_{aq}^{ - } + [R_{3} R^{\prime}PCl]_{org} \leftrightarrow [InCl_{4} \cdot R_{3} R^{\prime}P]_{org} $$

where R_3_R′R_+_  = tetradecyl-(trihexyl)phosphonium; A = bis-(2,4,4-trimethylpentyl)phosphinate).

### Artificial neural network procedure

**Figure 7:**
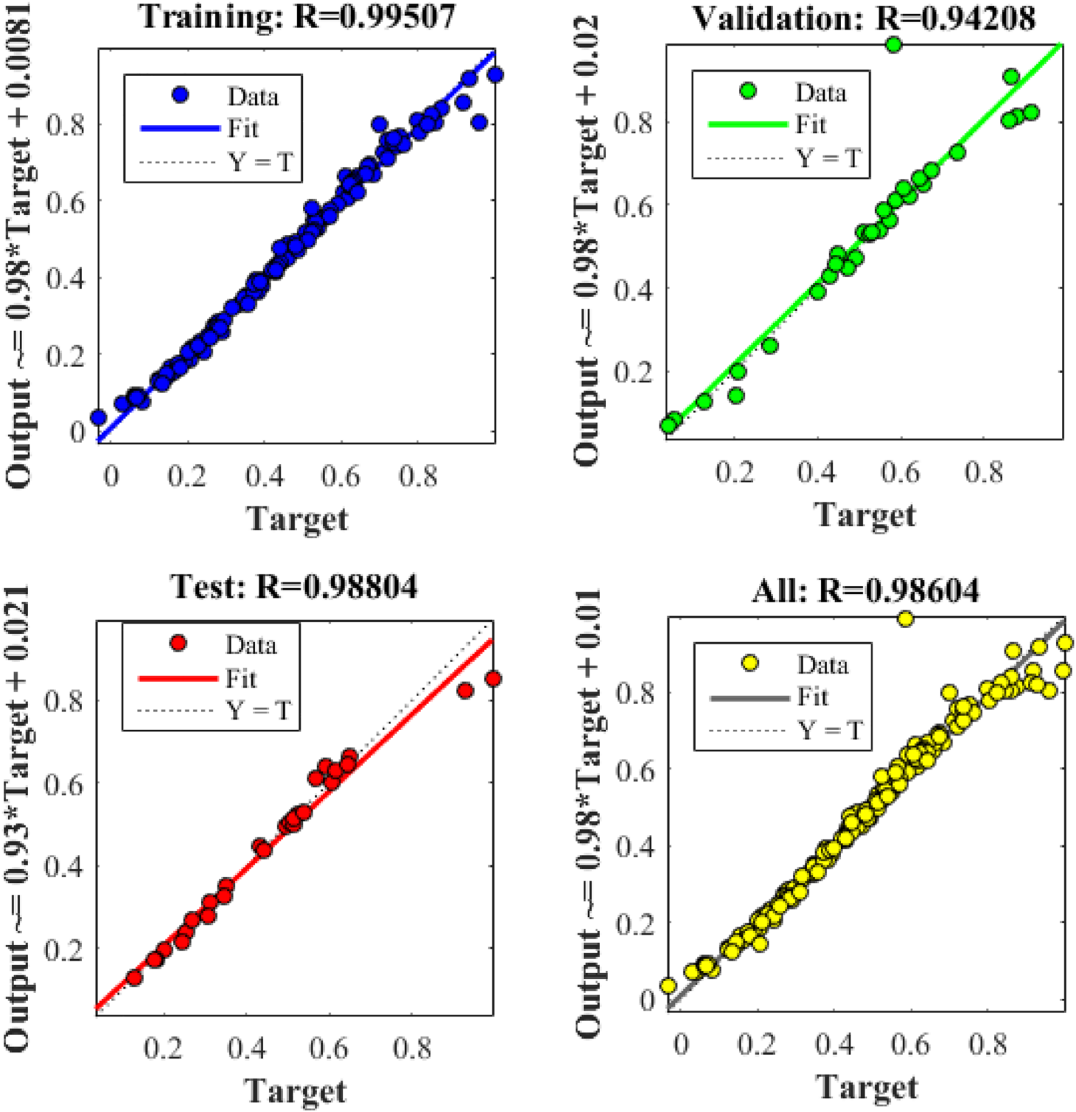
Regression plot of for three groups of training, validation, test and all data of artificial neural network with six hidden layer.

**Figure 8:**
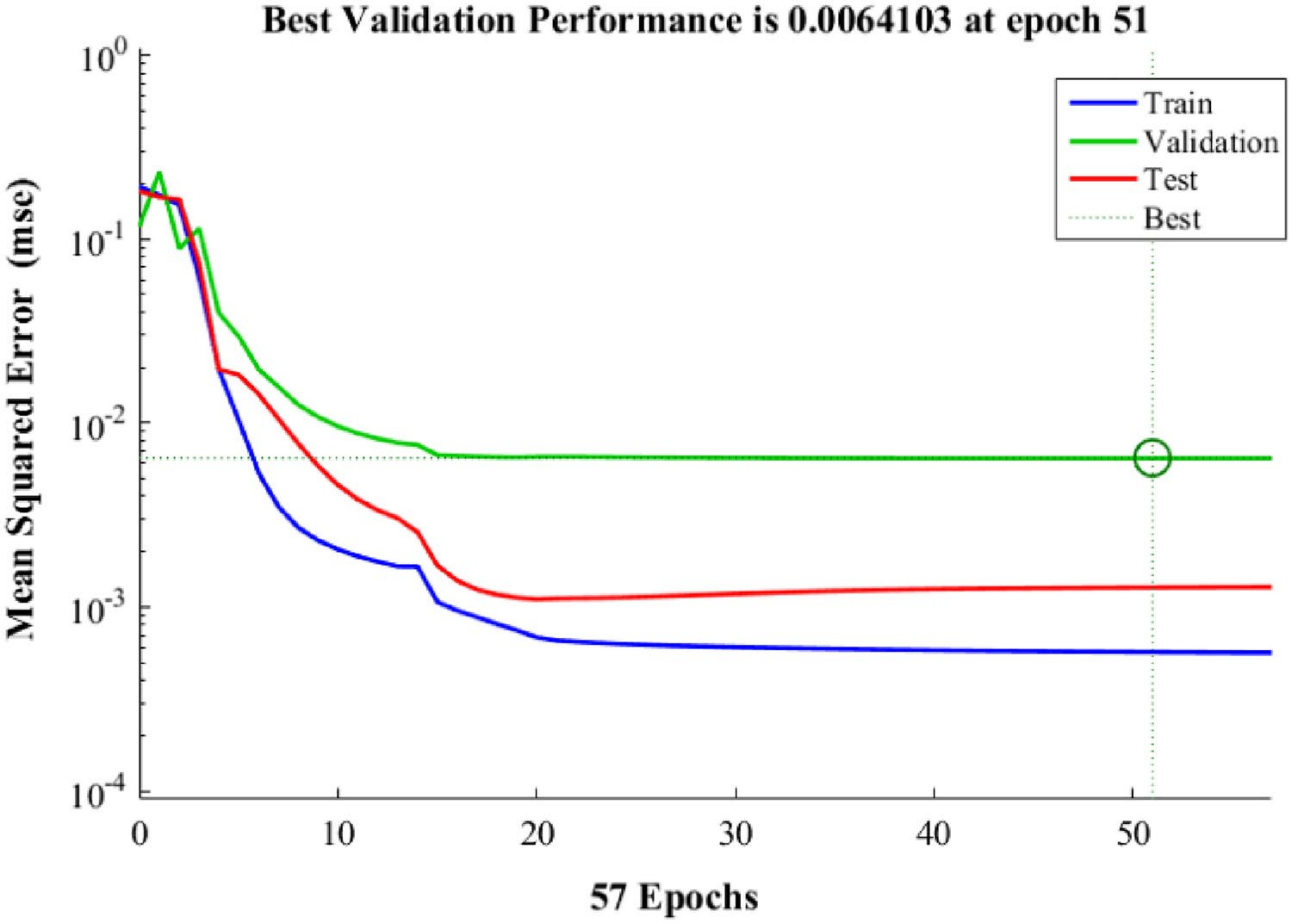
Validation performance of ANN model.

The variation in the number of neurons in middle layer of ANN design was carried out to reach the best results. The selection of number was evaluated the statistical analysis (least mean square error (MSE), high coefficient of determination (R^2^)), as shown in Table 4. The best predicted data was obtained with six neurons, as described in Fig. 7 (R^2^ = 0.9860 ~ total data, 0.9951 ~ training data 0.9421 ~ validation, and 0.9880 ~ testing). The AARE ~ 8.1127, MSE ~ 0.0011, and RMSE ~ 0.0245 are obtained with ANN approach. Also, lower data including AARE ~ 9.5848, MSE ~ 19.5679, and RMSE ~ 4.4237 for RSM approach described the minimum error in the application of ANN modeling. The optimum encapsulation of validation equal to 0.0064103 at epoch 51 was shown in Fig. 8.

**Table 4:** Performance of diverse networks with different numbers of neurons in the hidden layer.

Neuron number in hidden layer	Mean square error	R^2^ training	R^2^ validation	R^2^ testing
1	0.01867	0.83541	0.79634	0.89757
2	0.02435	0.80550	0.76468	0.96571
3	0.00842	0.96978	0.95659	0.95252
4	0.01820	0.87803	0.84109	0.70150
5	0.01482	0.91178	0.90564	0.98508
6	0.00509	0.96169	0.97247	0.99624
7	0.00571	0.96101	0.96329	0.92561
8	0.00565	0.98881	0.94856	0.86381
9	0.01267	0.90071	0.79128	0.81064
10	0.03097	0.99308	0.70048	0.78766
11	0.05663	0.93306	0.77279	0.73856
12	0.01273	0.85377	0.92284	0.82844

**Figure 9:**
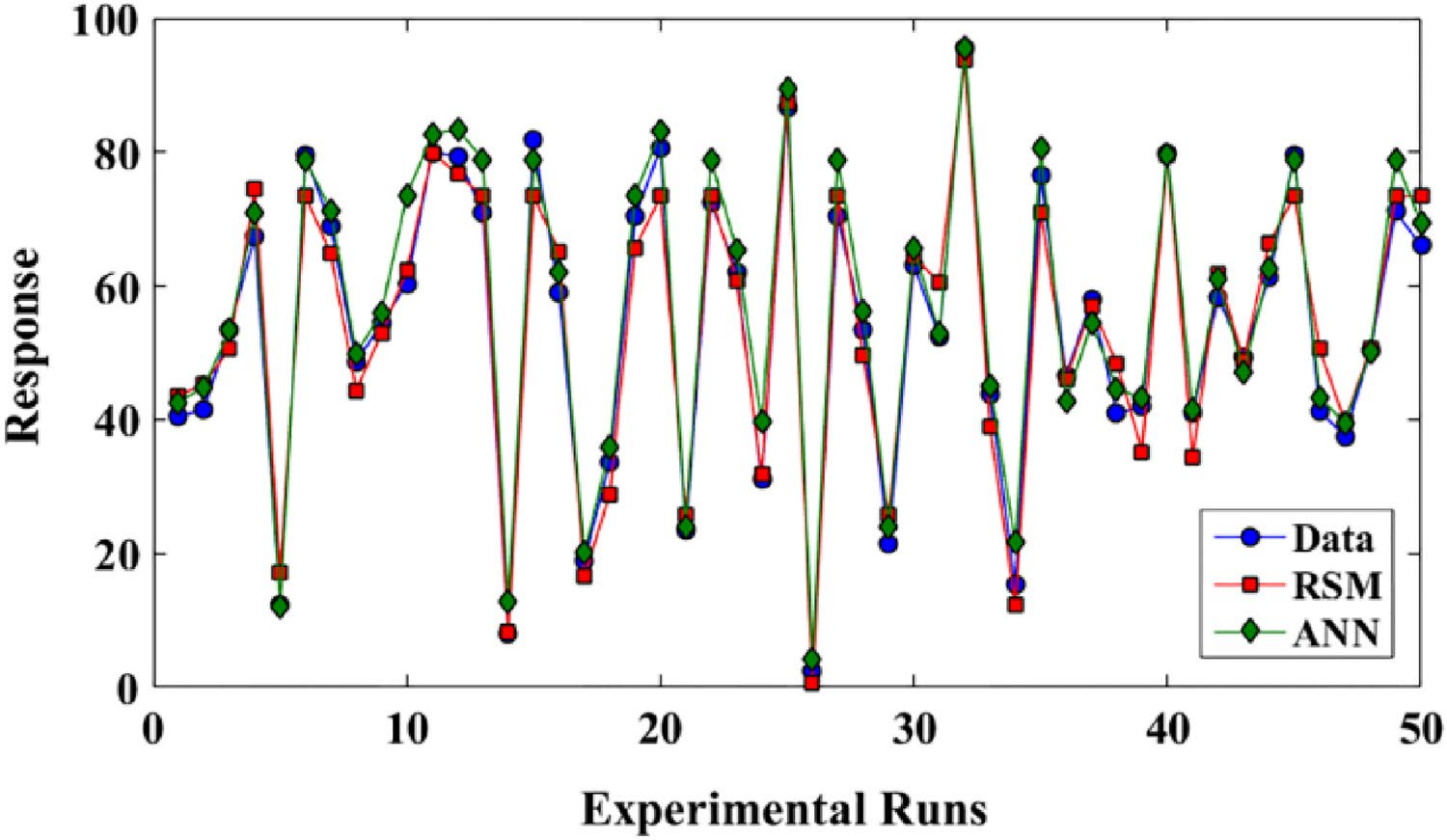
Comparison of obtained results from ANN and RSM predicted values.

The comparison of experimental data with the ANN and RSM models is shown in Table 5 and Fig. 9. The results illustrated the minimum errors with the ANN procedure compared to RSM model.

**Table 5:** Comparison of experimental data with the predicted values from ANN and RSM models.

Runs	HNO_3_ concentration (mol/L)	Indium concentration (mg/L)	Cyphos IL 104 concentration (mol/L)	Stripping concentration (mol/L)	Chloride concentration (mol/L)	Transport efficiency (%)		
						Experimental data	RSM data	ANN data
1	2.13	73.92	0.09	3.58	1.51	40.56	43.57	42.58
2	0.88	73.92	0.09	3.58	2.99	41.44	45.29	44.90
3	0.88	36.08	0.16	1.52	2.99	53.44	50.65	53.32
4	0.88	36.08	0.16	3.58	2.99	67.32	74.56	70.89
5	0.88	73.92	0.09	1.52	1.51	12.33	17.24	12.18
6	1.51	55.00	0.13	2.55	2.25	79.56	73.41	78.93
7	2.13	73.92	0.16	1.52	2.99	68.9	64.75	71.25
8	3.00	55.00	0.13	2.55	2.25	48.66	44.39	49.85
9	2.13	36.08	0.16	1.52	1.51	54.33	52.87	55.99
10	2.13	36.08	0.16	1.52	2.99	60.33	62.35	73.58
11	2.13	36.08	0.16	3.58	2.99	79.86	79.94	82.72
12	1.51	55.00	0.13	2.55	4.00	79.44	76.65	83.41
13	1.51	55.00	0.13	2.55	2.25	70.87	73.41	78.93
14	0.88	36.08	0.09	1.52	1.51	7.88	8.34	12.79
15	1.51	55.00	0.13	2.55	2.25	81.77	73.41	78.93
16	0.88	36.08	0.16	3.58	1.51	58.98	65.11	62.16
17	0.01	55.00	0.13	2.55	2.25	18.98	16.56	20.10
18	1.51	55.00	0.05	2.55	2.25	33.56	28.80	35.80
19	2.13	36.08	0.16	3.58	1.51	70.33	65.59	73.58
20	0.88	73.92	0.16	3.58	1.51	80.55	73.36	83.05
21	0.88	73.92	0.09	1.52	2.99	23.44	25.81	24.09
22	1.51	55.00	0.13	2.55	2.25	72.55	73.41	78.93
23	1.51	10.00	0.13	2.55	2.25	62.11	60.90	65.43
24	0.88	73.92	0.09	3.58	1.51	31.09	31.85	39.71
25	0.88	73.92	0.16	3.58	2.99	86.55	87.49	89.49
26	1.51	55.00	0.13	0.10	2.25	2.43	0.59	4.14
27	1.51	55.00	0.13	2.55	2.25	70.32	73.41	78.93
28	1.51	55.00	0.13	2.55	0.50	53.44	49.54	56.19
29	0.88	36.08	0.09	3.58	1.51	21.56	25.71	24.06
30	2.13	36.08	0.09	3.58	2.99	63.11	64.38	65.51
31	2.13	73.92	0.16	3.58	1.51	52.33	60.54	52.91
32	1.51	55.00	0.20	2.55	2.25	95.66	93.72	95.47
33	1.51	55.00	0.13	5.00	2.25	43.76	38.90	45.16
34	0.88	36.08	0.09	1.52	2.99	15.33	12.22	21.61
35	1.51	100.00	0.13	2.55	2.25	76.55	71.05	80.56
36	0.88	36.08	0.16	1.52	1.51	46.55	46.07	42.80
37	0.88	73.92	0.16	1.52	1.51	57.88	57.07	54.39
38	2.13	36.08	0.09	1.52	2.99	40.88	48.46	44.67
39	2.13	73.92	0.09	1.52	1.51	41.99	35.27	43.18
40	2.13	73.92	0.16	3.58	2.99	79.88	79.58	79.68
41	0.88	36.08	0.09	3.58	2.99	41.08	34.46	41.41
42	2.13	73.92	0.09	3.58	2.99	58.33	61.91	60.93
43	2.13	73.92	0.09	1.52	2.99	49.44	48.75	47.07
44	0.88	73.92	0.16	1.52	2.99	61.22	66.34	62.56
45	1.51	55.00	0.13	2.55	2.25	79.55	73.41	78.93
46	2.13	73.92	0.16	1.52	1.51	41.33	50.57	43.32
47	2.13	36.08	0.09	1.52	1.51	37.55	39.67	39.46
48	2.13	36.08	0.09	3.58	1.51	50.33	50.73	50.15
49	1.51	55.00	0.13	2.55	2.25	71.11	73.41	78.93
50	1.51	55.00	0.13	2.55	2.25	65.99	73.41	69.37

The particle swarm optimization was used for the determination of the optimum point by the ANN model. The best-optimized data were: 73.92 mg/L, 0.163 mol/L, 1.397 mol/L, 3.04 mol/L, and 3.00 mol/L for indium concentration, carrier concentration, feed phase acidity, chloride ion concentration, and stripping agent concentration. The results achieved by RSM and ANN led to an experimentally determined extraction efficiency of 93.91%, and 94.85%, respectively. The optimum point predicted by both RSM and ANN shows close agreement between the experimental data (95.77%) and the predicted values. Therefore, the obtained models are adequate to optimize the recovery of indium ions. Both RSM and ANN can be concluded as the appropriate models to use for the prediction and optimization process.

### Evaluation of indium extraction from the discarded LCD screen

**Figure 10:**
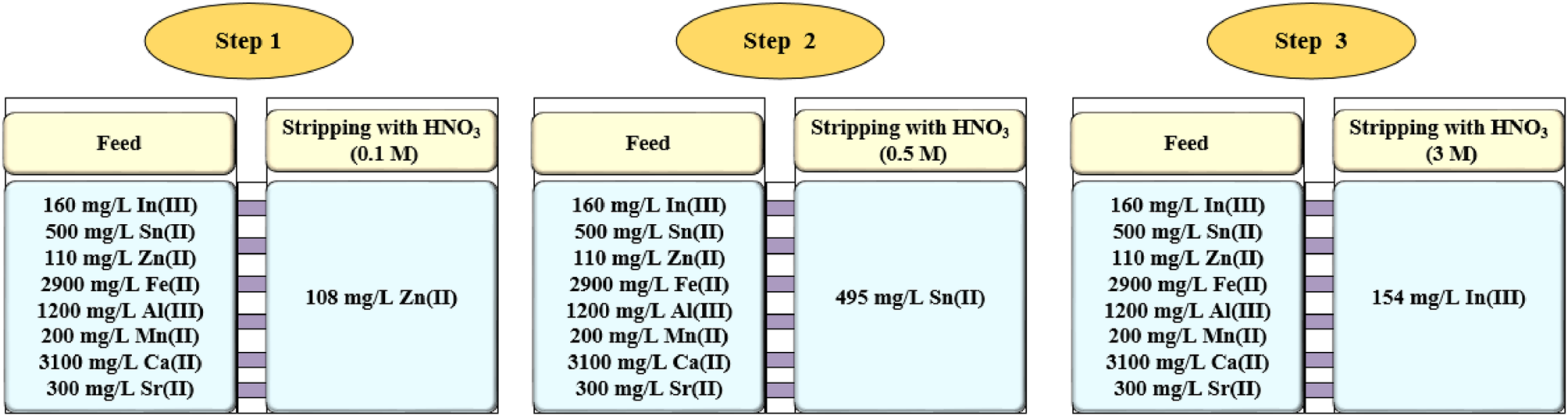
Procedure of indium extraction from the discarded LCD screen.

The synthesis solution (leaching solution of the discarded LCD screen) was used in the experiments. The concentration of metal ions in the synthesis solution are equal to 160, 500, 110, 2900, 1200, 200, 3100, 300 ppm for In, Sn, Zn, Fe, Al, Mn, Ca, Sr, respectively, according to the research of^64^. The zinc and tin ions are transferred to the membrane phase along with indium ions (efficiency higher than 91.5%). The other elements remained in the feed phase with an efficiency lower than 5%. The procedure of the indium extraction is shown in Fig. 10. Therefore, a weak acid solution of HNO_3_ ~ 0.1 M was used to strip zinc ions from the membrane phase (%99.71). In the second stage, the strip phase was replaced with a stronger acid (HNO_3_ ~ 0.5 M). In this stage, the tin ions were scrubbed from the membrane phase (%99.10). In the final stage, 3 M of HNO_3_ solution was used to strip indium with an efficiency higher than 95.77%.

## Conclusion

This research employed two modeling approaches for the recovery of indium ions using an ionic liquid. The Cyphos IL 104 was utilized as a carrier phase in a flat sheet supported liquid membrane to extract In(III) ions. The study investigated and modeled the impact of various factors, including the acidity of the feed phase, the concentration of In(III) ions, the concentration of the ionic liquid, the concentration of the stripping agent, and the concentration of chloride ions, on the efficiency of the extraction process using two methods: response surface methodology (RSM) and artificial neural network (ANN). The optimal values obtained were as follows: 73.92 mg/L for indium concentration, 0.157 mol/L for carrier concentration, 1.386 mol/L for feed phase acidity, 2.99 mol/L for chloride ion concentration, and 3.06 mol/L for stripping agent concentration. The extraction efficiency determined through the RSM and ANN methods was found to be 93.91% and 94.85% respectively, which closely matched the experimental data (95.77%). These results highlight the Cyphos IL 104 as a promising carrier phase for the liquid membrane extraction of In(III) ions with minimal solvent usage during the three stages of extraction and stripping from E-waste. This approach offers a potential means of intensifying the process to achieve maximum efficiency.

## Data Availability

The datasets used and/or analysed during the current study available from the corresponding author on reseanable request.
